# The efficacy and safety of ^225^Ac-PSMA-617 in metastatic castration-resistant prostate cancer

**DOI:** 10.3389/fonc.2025.1516860

**Published:** 2025-02-28

**Authors:** Jiao Ma, Yu Zhang, Jiangchu Yangqing, Guangfu Liu, Junzheng Wang, Chunyin Zhang

**Affiliations:** ^1^ Department of Nuclear Medicine, Affiliated Hospital of Southwest Medical University, Luzhou, Sichuan, China; ^2^ Nuclear Medicine and Molecular Imaging Key Laboratory of Sichuan Province, Southwest Medical University, Luzhou, Sichuan, China; ^3^ Laboratory for Targeted Radiopharmaceuticals Creation, Southwest Medical University, Luzhou, Sichuan, China; ^4^ Institute of Nuclear Medicine, Southwest Medical University, Luzhou, Sichuan, China

**Keywords:** prostate cancer, 225 Ac-PSMA-617, radioligand therapy, 68 Ga-PSMA, PET/CT

## Abstract

**Background:**

We aimed to report our clinical experience with the use of ^225^Ac-PSMA-617 in the treatment of mCRPC patients.

**Methods:**

A retrospective analysis was conducted on 29 metastatic castration-resistant prostate cancer (mCRPC) patients treated with ^225^Ac-PSMA-617. Patients underwent treatment at 8-week intervals and discontinued treatment upon disease progression or the occurrence of intolerable adverse effects. We acquired ^68^Ga-PSMA-11 PET/CT images and laboratory test outcomes of patients at baseline and 8 weeks following each treatment. Short-term efficacy was evaluated through the biochemical response of serum prostate-specific antigen (PSA) and molecular tumor response criteria. A follow-up was conducted to assess the long-term effectiveness by examining the patient’s overall survival (OS) and progression-free survival (PFS). The numerical rating scale (NRS) assessed the patient’s pain. The side effects after treatment were evaluated based on common terminal criteria for adverse events version 5.0 (CTCAE v5.0).

**Results:**

29 patients with mCRPC underwent a total of 50 treatment cycles. The median age of the patients was 67 years (55-84years). Out of these patients, 11 had previously underwent ^177^ Lu-PSMA-617 radioligand therapy (RLT). After treatment, any PSA decline was observed in 75.9% of patients, and a PSA decline≥50% was observed in 62.1%. 61.1% of patients had disease control according to molecular response. The estimated OS and PFS were 18 months (95% CI: 15-21 months) and 8 months (95% CI: 6-10 months). Univariate analysis showed that any PSA decline was positively correlated with PFS (p<0.001). The most common side effect was xerostomia, observed in 79.3% of patients. Grade III blood toxicity was observed in 7/29 patients. After treatment, the pain disappeared in 4 patients and was relieved in 13 individuals.

**Conclusions:**

In mCRPC, the results indicated that ^225^Ac-PSMA-617 demonstrated a favorable disease control rate and relatively minimal side effects. However, additional high-quality randomized controlled trials are required for future validation.

## Introduction

Prostate Cancer (PCa) is one of the most common cancers in the urogenital system of men worldwide. The 2020 global cancer statistics report reveals that prostate cancer is the third most prevalent cancer, following lung cancer and colorectal cancer. Among cancers affecting men globally, prostate cancer ranks second in terms of incidence rate and fifth in terms of fatality rate (1). The early symptoms are not obvious. When diagnosed, patients are often in the late stage (2). Androgen deprivation therapy (ADT) is effective for treating advanced prostate cancer. However, patients may develop castration resistance after a 1-2 year period of being sensitive to androgen (3, 4). This resistance can cause the disease to progress rapidly, accelerate metastasis, and ultimately lead to metastatic castration-resistant prostate cancer (mCRPC) (5). Chemotherapy (such as docetaxel and cabazitaxel), androgen receptor signaling inhibitors (ARSI) (such as Abiraterone, enzalutamide, or apalutamide), poly adenosine diphosphate ribose polymerase (PARP) inhibitors, immunotherapy have been used for mCRPC patients (6, 7). Although these drugs are used, as the patient’s condition progresses, the efficacy of these therapies may gradually decrease or even become completely ineffective (8).

Targeted Radionuclide Therapy (TRNT) has become a pivotal therapeutic strategy in oncology due to its precise targeting capability towards tumor cells (9). In recent years, the radioactive labeled Prostate Specific Membrane Antigen (PSMA) ligand has been used to diagnose and treat prostate cancer and has achieved good results (10, 11). Its expression level in prostate cancer tissue increased by about 100-1000 times when compared with normal prostate tissue, and the expression level is much higher in the poorly differentiated, metastatic-castration-resistant PCa tissues (12). PSMA-617 is a potent PSMA inhibitor with a strong binding affinity. As a compassionate therapy, ^225^Ac-PSMA-617 has been proven beneficial in patients with advanced mCRPC who have not responded to or have continued to worsen after receiving ^177^Lu-PSMA-617 RLT (13, 14). Compared with ^177^Lu, ^225^Ac has higher radiation energy, thus having more significant potential in inducing DNA double helix breakage and cell killing in the nucleus. In addition, ^225^Ac-PSMA-617 also has the advantage of targeting any metastatic tissue and has good application prospects for small, scattered, and micro-metastatic lesions (15, 16). This article aims to summarize the data from our research center and provide clinical evidence and experience for treating mCRPC with ^225^Ac-PSMA-617 worldwide.

## Materials and methods

### Patients

A retrospective study was conducted on mCRPC patients who underwent ^225^Ac-PSMA-617 radioligand therapy at our hospital from July 2021 to December 2023. This study adhered to the ethical principles outlined in the Helsinki Declaration and received approval from our Ethics Review Committee (AHSWMU-2020-035). Patients and their families willingly consented to the therapy and completed an informed consent form. Inclusion criteria: ① Patients were diagnosed with mCRPC. ② The patients’ life expectancy was ≥ 6 months.

③ ^68^Ga-PSMA PET/CT showed the expression of PSMA in the lesions was higher than in the liver at baseline. ④ Eastern Cooperative Oncology Group(ECOG)score was below 4 points. Exclusion criteria: ① Severe bone marrow suppression: Hb<60g/L, PLT<25×10^9^g/L, WBC<3×10^9^g/L. ② Poor liver and kidney function: ALB ≤ 25g/L, bilirubin ≥ 1.5 times the upper limit of normal value; Creatinine ≥ 2 times the upper limit of normal value GFR ≤ 30ml/min/1.73m^2^.

### Preparation

According to the recommendation of the Prostate Cancer Clinical Trials Working Group (PCWG3) (17), patients planning to receive ^225^Ac-PSMA-617 treatment undergo laboratory tests within one week before treatment, including serum PSA, Hemoglobin (Hb), White blood cell (WBC), Platelets (PLT), Albumin (ALB), Alanine aminotransferase (ALT), Aspartate aminotransferase (AST), Total bilirubin (TBIL), Creatinine (Cr), and Glomerular Filtration Rate (GFR), Alkaline Phosphatase (ALP), Lactate Dehydrogenase (LDH), etc. Collected baseline ^68^Ga-PSMA-11 PET/CT images within one week before treatment. After administering 1.85 MBg/kg of ^68^Ga-PSMA via intravenous injection, patients underwent a whole-body PET/CT scan (uM789 PET/CT) 40-60 minutes later. Subsequently, we assessed the presence of PSMA in tumor lesions by measuring the maximum standard uptake value (SUVmax) and the peak standard uptake value of lean body weight (SULpeak).

### Synthesis of ^225^Ac-PSMA-617

The ^225^Ac was dissolved in 0.04 M hydrochloride acid (ITM, Germany). The PSMA-617 was obtained from ABX (Germany). A 0.1 M sodium ascorbate solution (100 mg sodium ascorbate dissolved in 5 mL MQ of water) was used as a buffer. ^225^AcCl_3_ was added to the reaction mixture of PSMA-617 (100μg) with buffer and heated for 25 minute at 100°C. The radiochemical purity of the products was analyzed by high-performance liquid chromatography. Products with a radiochemical purity of >99% were injected into the patients.

### Administration of ^225^Ac-PSMA-617

To prevent vomiting, the patients were instructed to take ondansetron oral soluble pellicles orally at least 30 minutes before the infusion of amino acids. Ondansetron was continuously taken orally at a dose of 8 mg, 3 times a day, for 3 consecutive days after the treatment. After 30 minutes of infusion of physiological saline, ^225^Ac-PSMA-617 was slowly administered via intravenous micropump (10-20 minutes). The dosage of ^225^Ac-617-PSMA was 200 μCi, and the interval between each treatment was 8 weeks.

### Treatment response

Blood routine, liver and kidney function, and serum PSA were measured at 2, 4, and 8 weeks after each treatment as routine follow-up; ^68^Ga-PSMA PET/CT imaging was performed 8 weeks after each treatment cycle to evaluate molecular response.

Then, the biochemical response was evaluated by detecting changes in serum PSA relative to baseline. Molecular response was performed by Response Evaluation Criteria in Solid Tumors 1.1 (RECIST 1.1) and PET Response Evaluation Criteria in Solid Tumors 1.0 (PERCIST 1.0) based on ^68^Ga-PSMA PET/CT for imaging analysis of lesions to evaluate their therapeutic response (18, 19). Disease control was defined as the absence of disease progression over a certain period of time, encompassing both disease stability and disease response. Disease stability included Stable Disease (SD) based on RECIST 1.1 and Stable Metabolic Disease (SMD) based on PERCIST 1.0. Disease response included disease response (PR) based on RECIST 1.1 and Partial Metabolic Response (PMR) based on PERCIST 1.0. The Numeric Rating Scale (NRS) was used to evaluate the pain in prostate cancer patients with bone metastasis after treatment (20). A score of 0-10 represents varying degrees of pain. 0 points: painless, 1-3 points: light pain, 4-6 points: moderate pain, 7-10 points: severe pain.

### Safety

All treatment-related side effects were defined and graded according to CTCAE 5.0 (21) during the baseline and follow-up periods.

### Statistical analysis

IBM SPSS 24.0 software was used for statistical analysis. A paired sample t-test/Wilcoxon signed-rank test was used to determine the differences in laboratory tests before and after treatment. Waterfall plots were used to display the percentage change in PSA levels relative to baseline at different time points. The pain levels before and after therapy were assessed using Fisher’s exact test for precision probability. Kaplan-Meier survival curve and log- rank test were used to estimate PFS and OS. Univariate analysis was performed to identify potential factors influencing survival, P<0.05 is considered statistically significant.

## Results

### Patients characteristics

29 mCRPC patients (median age:67 years; range: 55-84 years) were included in the study. Eleven patients were over 70 years old, and 4 patients had a family history of PCa. The median time from diagnosis of PCa to initiation treatment with ^225^Ac was 47 months (11-178 months). Baseline PSA level was 124.33ng/mL(6.25-1717.18ng/mL).The median treatment cycles were 2 (1-4). Eleven patients received 1 cycle, 12 received 2 cycles, 1 received 3 cycles, and 3 received 4 cycles. More detailed baseline patient characteristics are provided in **Table 1**.

**Table 1:** Patient characteristics.

characteristics	Value
No. of patients (n)	29
Median age (yrs)	67 (55-84)
Age ≥ 70 years, n (%)	11 (38.0%)
Family history of PCa, n (%)	4 (13.8%)
Time to start treatment from initial diagnosis of PCa, (mths)	47 (11-178)
ECOG score, n (%)	
0	7 (24.1%)
1	13 (44.8%)
2	9 (31.1%)
Gleason score at initial diagnosis, n (%)	
7	9 (31.1%)
8	4 (13.8%)
9	13 (44.8%)
10	3 (10.3%)
Treament cycle	2 (1-4)
Baseline PSA level,(ng/mL)	124.33 (6.25-1717.18)
Sites of metastases, n(%)	
Bone	28 (96.6%)
Lymph node	17 (58.6%)
Lung	8 (27.6%)
Liver	4 (13.8%)
Brain	2 (6.9%)
Other organs	8 (27.6%)
Previous therapies n(%)	
Prostatectomy	8 (27.6%)
ADT	29 (100%)
Radiation	9 (31.1%)
Chemotherapy Docetaxel/Cabazitaxel	21 (72.4%)
Abiraterone	14 (48.3%)
Enzalutamide or and Apatamide or and Darotamide	10 (34.5%)
Targeted therapy/immunotherapy	12 (41.4%)
^177^Lu-PSMA RLT	11 (37.9%)

yrs, years; n, number; mths, months; Pca, Prostate Cancer; ECOG, Eastern Cooperative Oncology Group; PSA, prostate- specific antigen; ADT, Androgen Deprivation Therapy; RLT, Radioligand Therapy.

### Treatment response

#### Biochemical response

**Figure 1:**
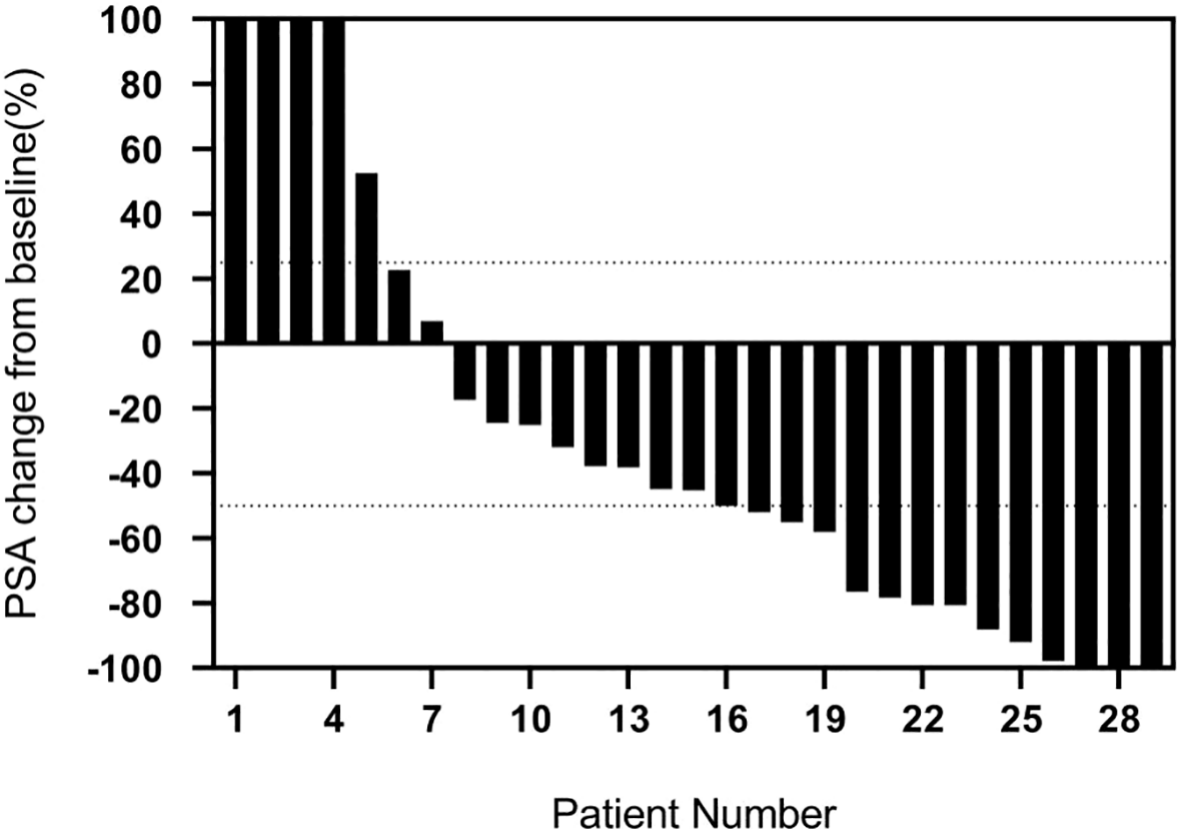
Percentage change in PSA after the first cycle of ^225^Ac-PSMA-617 (N=29).

**Figure 2:**
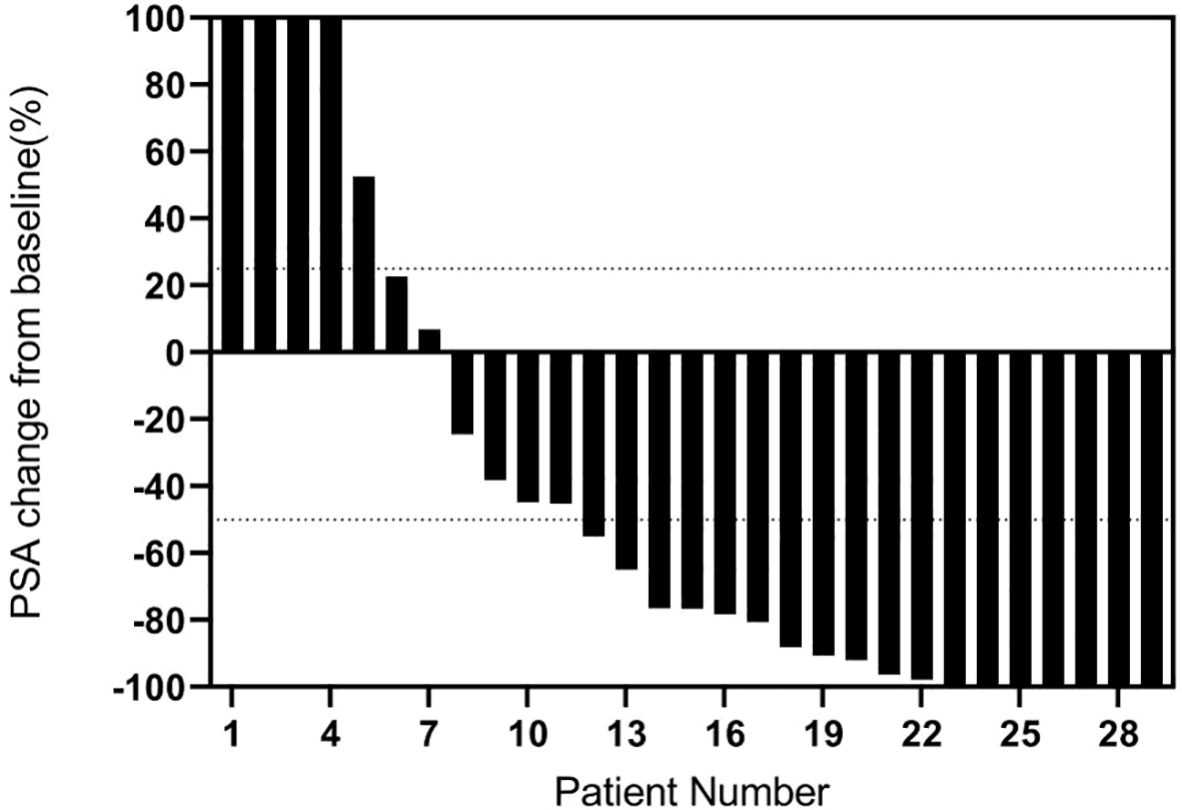
Percentage change in the best PSA after ^225^Ac-PSMA-617 (N=29).

Any PSA decline was seen in 75.9%(22/29) of patients two months after the first cycle of treatment, with PSA decline ≥ 50% in 48.3% (14/29) of patients and PSA increase in 24.1% (7/29) of patients, as shown in **Figure 1**. After the second treatment cycle, a decrease in PSA levels was observed in 81.2% (13/16) of patients. Among these patients, 75% (12/16) experienced a PSA fall of at least 50%. Conversely, 18.8% (3/16) of patients had increased PSA levels. Three of the four patients showed a decrease in PSA levels following the third treatment cycle. Specifically, two patients revealed a 50% or more fall in PSA levels, whereas one patient showed an increase in PSA levels. Finally, three patients had PSA decline after the fourth treatment cycle, and two had PSA decline ≥ 50%. Among the 7 patients with a PSA increase, 4/7 had previously undergone 1-2 cycles of ^177^Lu-PSMA RLT. In all treatment cycles, 75.9% of patients had any PSA decline, and 62.1% had PSA decline ≥ 50%. 63.6% (7/11) of patients who were exposed to ^177^Lu-PSMA RLT before had any PSA decline, while 45.5% (5/11) had a PSA decline ≥ 50%. Among the ¹⁷⁷Lu-PSMA RLT-naive patients, 82.4% (14/17) had any PSA decline, and 70.6% (12/17) had PSA decline≥ 50%. The optimal response to PSA is shown in **Figure 2**.

#### Molecular response

**Figure 3:**
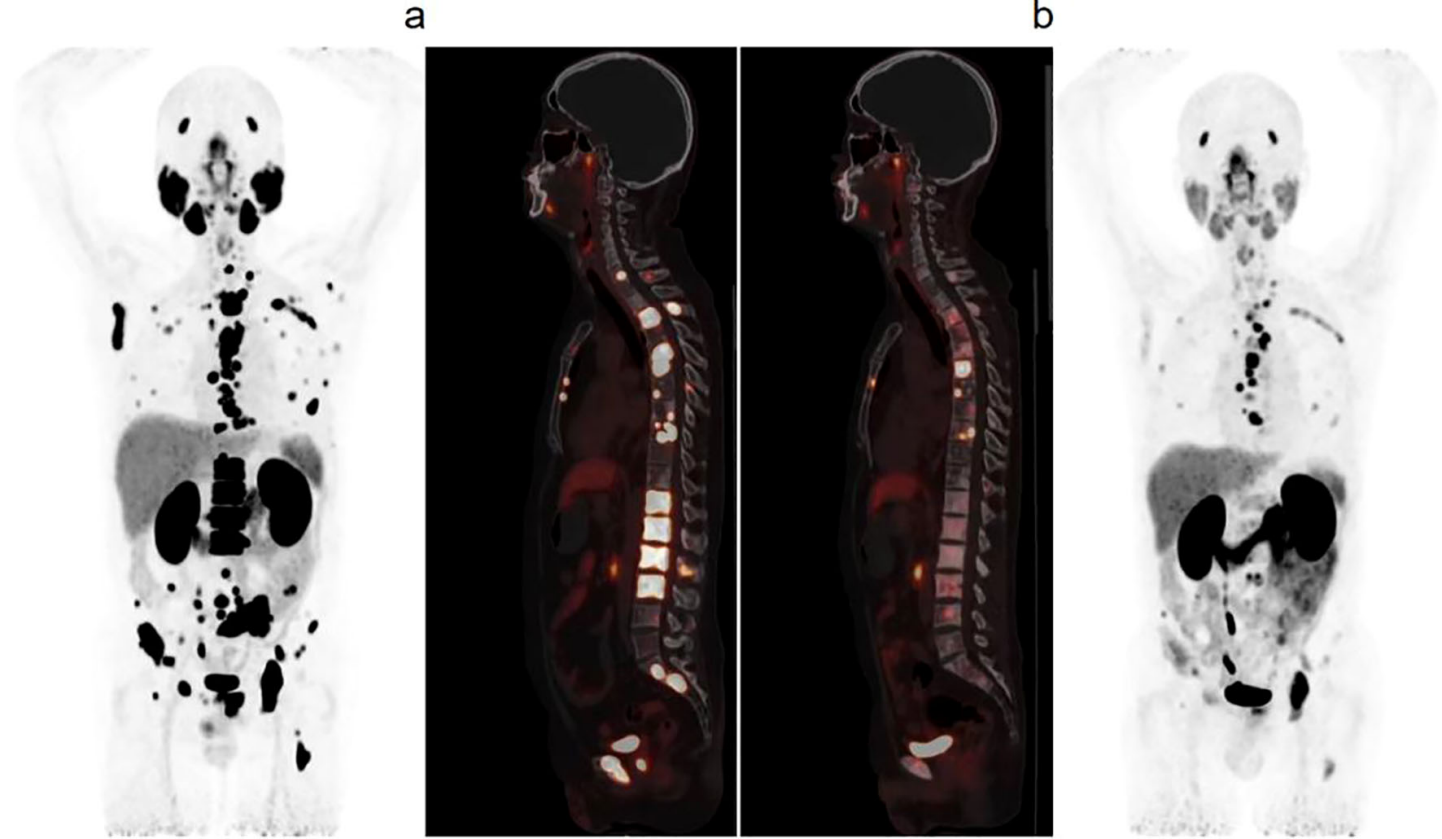
After 3 cycles of ^225^Ac-PSMA-617 treatment, the patient’s PSA decreased from 601.326 ng/ml to 7.486ng/ml. Compared to the baseline, ^68^Ga-PSMA-11 PET/CT images showed a significant decrease in tracer uptake in multiple metastases throughout the body after treatment **(a, b)**.

^68^Ga-PSMA-11 PET/CT images of 29 patients were acquired at baseline. But pre- and post-treatment ^68^Ga-PSMA-11 PET/CT images were only acquired for 18 patients. Out of the total of 18 patients, 11 (61.1%) were found to be under disease control, as indicated by **Figure 3**. Of the 18 patients, 14 (77.7%) showed consistent evaluation results according to RECIST 1.1 and PERCIST 1.0. During PET/CT follow-up two months after the last therapy, seven patients (38.9%) were classified as having progressive disease (PD) or progressive metabolic disease (PMD) (new lesions and increasing PSMA expression in lesions), 3 patients (16.7%) were assessed as stable disease (SD)/stable metabolic disease (SMD), and 4 patients (22.2%) were evaluated as partial response (PR)/partial metabolic response (PMR). The evaluation results of RECIST 1.1 and PERCIST 1.0 in 4 patients were inconsistent and assessed as SD/PMR (**Supplementary Table 5**).

### Survival period

As followed up, 13 patients died. The median OS was 18 months (95% CI: 15-21 months); The median PFS was 8 months (95% CI: 6-10 months) (**Supplementary Figures 4**, **5**). The median PFS between ¹⁷⁷Lu-PSMA RLT-exposed patients and ¹⁷⁷Lu- PSMA RLT-naive patients was 5.3 months (95% CI: 2.5-8.1months) versus 13.6 months (95% CI: 10.1-17.0 months) (p=0.004). The median OS was 16.7 months (95% CI: 12.9-20.6 months) versus 16.7 months (95% CI: 15.0-18.4 months) (p=0.84). Univariate analysis correlated any PSA decline and PFS (p<0.001) (**Supplementary Figure 6**). The median PFS of patients with any PSA decline was 10 months (95% CI: 3-17 months), and the median PFS of patients with PSA increase was 2 months (95% CI: 3-17 months). In 18 patients, the median PFS of PMD and SMD/PMR was 4.3 months (95% CI: 2.6-6.0 months) versus 14.3 months (95% CI: 10.8-18.0 months) (p<0.001)(**Supplementary Figure 7**).

### Pain assessment

Out of the 23 patients, 3 patients were pain-free before therapy, 3 experienced light bone pain, 7 experienced moderate bone pain, and 10 experienced severe bone pain. Two months after the last treatment, the pain of four patients completely disappeared, six patients experienced a transition from moderate to light pain, one patient was from severe to light pain, three patient was from severe to moderate pain, another six patient reported no change, as shown in **Table 2**.

**Table 2:** The pain at baseline and after ^225^Ac-PSMA-617 according to numerical rating scale.

	baseline	after ^225^Ac-PSMA-617	p
0	3	7	0.133
1-3	3	7	
4-6	7	3	
7-10	10	6	

### Toxicity

During follow-up, the most common toxicity among patients was xerostomia; 79.3% (23/29) of patients had xerostomia. 62.1% (18/29) of patients developed I-II grade xerostomia after treatment. Five patients had previously received ^177^Lu-PSMA RLT and had grade II xerostomia. After this treatment, they progressed to grade III. Following treatment, nine patients experienced grade I weight loss, one experienced grade I anorexia, four experienced grade I fatigue, three experienced constipation, two experienced insomnia, and one patient with indwelling catheters experienced grade I hematuria. However, in subsequent follow-up observation, the patient’s hematuria symptoms disappeared, as shown in **Table 3**.

**Table 3:** Therapy-related side effects after ^225^Ac-PSMA-617 according to CTCAE v5.0.

	Prior to ^225^Ac-PSMA-617 Therapy			After ^225^Ac-PSMA-617 Therapy		
	Grade I-II	Grade III	Grade IV	Grade I-II	Grade III	Grade IV
Anemia	22 (75.9%)	2 (6.9%)	–	20 (69.0%)	6 (20.7%)	–
Leucopenia	5 (17.2%)	–	–	8 (27.6%)	–	–
Thrombocytopenia	8 (27.6%)	–	–	6 (20.7%)	1 (3.4%)	–
Xerostomia	5 (17.2%)	–	–	18 (62.1%)	5 (17.2%)	–
Hypoalbuminemia	13 (44.8%)	–	–	15 (51.7%)	–	–
Fatigue	3 (10.3%)	–	–	7 (24.1%)	–	–
Constipation	4 (13.8%)	–	–	7 (24.1%)	–	–
Anorexia	5 (17.2%)	–	–	6 (20.7%)		–
Weight loss	–	–	–	9 (31.0%)	–	–
Insomnia	4 (13.8%)	–	–	6 (20.7%)	–	–
Hematuria	–	–	–	1 (3.4%)	–	–

There was no statistically significant difference in ALB, ALT, AST, ALB, TBIL, LDH, and ALP levels before and after treatment (p>0.05). After treatment, It was seen statistically significant decreases (p<0.05) in WBC, HB, Cr, and GFR (**Table 4**)(**Supplementary Figure 8**).

**Table 4:** Laboratory parameters at baseline and after ^225^Ac-PSMA-617.

	Baseline (mean±SD )	After 1st dose ^225^Ac-PSMA-617 (mean±SD )	After last dose ^225^Ac-PSMA-617 (mean±SD )	p
Leukocytes (G/l)	5.40±1.94	4.67±1.44	4.51+1.42	0.042
Hemoglobin (g/dl)	103.31±18.23	97.22±20.20	95.71±19.62	0.033
Platelets (G/l)	185.93±89.66	175.03±77.49	177.45±79.24	0.279
ALT (U/l)	17.1±9.73	18.94±13.06	19.97±13.96	0.252
AST (U/l)	27.81±13.68	29.41±20.98	30.81±21.31	0.443
Albumin (g/dl)	41.28±4.43	40.05±4.78	39.74±4.75	0.225
Bilirubin (mg/dl)	11.81±4.64	11.79±5.28	12.24±5.25	0.502
LDH (U/l)	305.62±193.63	281.79±183.59	275.17±188.76	0.058
ALP (U/l)	217.03±253.89	207.32±254.32	237.17±373,71	0.991
Creatinine (mg/dl)	70.61±20.09	70.01±21.57	69.98±22.13	0.01
eGFR (ml/min)	92.92±15.11	87.64±24.05	86.24±24.13	0.036

ALT, Alanine aminotransferase; AST, Aspartate aminotransferase; LDH, Lactate Dehydrogenase; ALP, Alkaline Phosphatase; eGFR, estimated Glomerular Filtration Rate.

No grade IV blood toxicity was found in all patients following therapy. Among patients with modest bone marrow suppression at baseline, 4 patients who had previously received ^177^Lu-PSMA RLT progressed to grade III anemia after this treatment, and 1 progressed to grade III thrombocytopenia. Out of the patients who had normal bone marrow at baseline, 2 patients developed mild anemia, 3 patients experienced a mild decrease in white blood cell count, and 2 patients experienced a mild reduction in albumin levels after treatment.

## Discussion

PSMA has recently emerged as an essential target for detecting and treating PCa due to its reasonable tissue selectivity. ^225^Ac-PSMA-617 shows promise for mCRPC. Our study presents the PSA response rate in a cohort of 29 patients with mCRPC. By comparing the changes in biomarkers before and after treatment, we found that the PSA level decreased significantly after treatment. When ^225^Ac-PSMA-617 induces apoptosis or necrosis of tumor cells, the amount of PSA produced and secreted by tumor cells also decreases accordingly, leading to a decline in the PSA level in the blood. Out of all the treatment cycles, a decrease in PSA levels was observed in 75.9% of the patients. A reduction of at least 50% in PSA levels was observed in 62.1% of patients, which was lower than the study conducted by Sathekge M et al. (22), which included 73 patients with mCRPC. They reported that 24 patients underwent 4-8 cycles of ^225^Ac-PSMA RLT. Nevertheless, aside from the diverse patient cohorts, only 3 patients in our study received 4 therapy cycles and the different treatments administered before may all caused the variation. Previous studies have shown that if an individual was previously exposed to ^177^Lu-PSMA RLT, then the ²²⁵Ac-PSMA RLT applied is associated with a lower PSA response rate and shorter time to death or disease progression (22–24). In our study, similar results were also observed. We compared the PSA response rate between ¹⁷⁷Lu-PSMA RLT-exposed patients and ¹⁷⁷Lu-PSMA RLT-naive patients. We find the PSA response was lower in the patients with previous exposure to ¹⁷⁷Lu-PSMA RLT than the ¹⁷⁷Lu-PSMA RLT-naive patients, 63.6% versus 82.4%. The result of PSA decline≥ 50% was 45.5% versus 70.6%, and it had a similar result to the study by Yadav et al. which was 26.6% versus 53.8% (19). Besides, among the patients assessed as PD/PMD by molecule response, 5/7 had previously received ^177^Lu-PSMA RLT. In our study, the median PFS of ¹⁷⁷Lu-PSMA RLT-exposed patients was lower than ¹⁷⁷Lu-PSMA RLT-naive patients, which was 5.3 months versus 13.6 months(p=0.004). But the median OS between them was of no difference. It may because the patients treated with ^177^Lu before had a higher disease aggressiveness and larger tumor burdens. In our study, two patients experienced PSA flicker, with a PSA increase after 1 treatment cycle. However, during follow-up, they reported that their bone pain had slightly improved, and their appetite had also improved. Therefore, we suggested they continue with ^225^Ac-PSMA-617 RLT. After the subsequent treatments, their bone pain was reduced, and PSA continued to decline (**Supplementary Table 6**, **Supplementary Figures 9**, **10**). The phenomenon of PSA flicker may be associated with tumor heterogeneity, treatment - induced cellular stress, changes in the tumor microenvironment, and the dynamic nature of PSA secretion (23, 25). A significant decrease in PSA level (e.g., a decrease of >50%) is generally considered a sign of effective treatment. However, the flicker phenomenon may impede the assessment of the condition and treatment outcome, resulting in an early drug stoppage or a change in treatment strategies. Consequently, the treatment response should be comprehensively evaluated by combining imaging (such as 68Ga-PSMA PET/CT) and clinical symptoms. When necessary, the duration of treatment ought to be suitably prolonged and evaluated in light of clinical symptoms.

According to molecular response, the efficacy evaluation results of 4 patients were inconsistent. In RECIST 1.1, the evaluation result was SD, while in PERCIST 1.0, the evaluation result was PMR. In contrast to PERCIST 1.0, RECIST 1.1 was based on CT anatomical imaging data, when there are no target lesions, the partial response of the tumor cannot be differentiated from stable or non-progression, Due to the widespread bone metastasis in mCRPC, PERCIST 1.0 based on ^68^Ga-PSMA PET/CT can evaluate the molecular biological changes of tumors through molecular imaging, which can more sensitively and accurately reflect the therapeutic effect of tumors in early treatment. Our study found that PERCIST 1.0 had a significant difference in distinguishing PFS between disease control (SMD/PMR) and disease progression(PMD)(p<0.001). Particularly in differentiating survival between individuals with good treatment responses and those with stable disease, PERCIST 1.0 could offer helpful prognostic information for mCRPC patients undergoing PSMA-617 RLT (19).

The efficacy of ^225^Ac-PSMA has been studied in mCRPC patients with different settings, who were treated with chemotherapy, hormone deprivation therapy, ^177^Lu-PSMA RLT, etc, even in patients with *de novo* hormone-sensitive prostate cancer (26–28). The results indicated that ^225^Ac-PSMA still remains highly effective. Recently, in a multicenter and retrospective study, 488 men with mCRPC received 1174 cycles of ^225^Ac-PSMA RLT (24). They reported the median PFS was 7.9 months. The median OS was 15.5 months. In our study, the median PFS was 8 months, and the median OS was 18 months. They found a PSA decline of ≥ 50% was associated with longer PFS and OS (p<0.05). Previous docetaxel or cabazitaxel/previous abiraterone or enzalutamide/previous abiraterone or enzalutamide/Anemia at baseline/patients with liver, peritoneal, or visceral metastases were all associated with shorter PFS and OS (p<0.05). However, our research analysis found that only any PSA decline was associated with longer PFS (p<0.001). Maybe the low patient numbers limited the findings. But this is a direction worthy of exploration and more controlled experiments are needed.

Following one to four treatment cycles, the pain was reported to have entirely vanished in four patients and eased in thirteen. The majority of patients with advanced prostate cancer have widespread bone metastases, which significantly lowers their quality of life and results in symptoms like discomfort, pathological fractures, and spinal cord compression. Some patients experienced varying degrees of improvement in pain symptoms after receiving ^225^Ac-PSMA-617 treatment. From the perspective of long-term follow -up, the duration and degree of pain relief may be influenced by multiple factors, such as tumor burden, treatment dose, and the patient’s own pain sensitivity. ^225^Ac-PSMA- 617 may relieve pain by targeting and killing tumor cells, thereby reducing the compression and invasion of surrounding tissues and nerves by the tumor. ^225^Ac-PSMA-617 targets the lesion with a short radiation range and no significant impact on surrounding tissues and organs, especially for patients with multiple bone metastases. It may significantly decrease bone pain and enhance the quality of life for the patient.

As PSMA is expressed in non-tumor organs like the salivary glands and kidneys, using PSMA-targeted therapy might cause radiation damage to the salivary glands that is irreversible and interferes with daily life for patients. During the subsequent monitoring, no grade IV or higher adverse effects were observed. The predominant adverse event seen was grade I-III xerostomia. Furthermore, the only symptoms seen were minor weariness, constipation, anorexia, sleeplessness, and weight loss. Studies have shown that for prostate cancer with widespread bone metastasis, the incidence of severe hematotoxicity after treatment with ^225^Ac-PSMA-617 was rare (29). The present investigation did not observe any cases of grade IV or higher blood toxicity. In a long-term follow-up study on renal toxicity after ^177^Lu-PSMA treatment, it was found that in most mCRPC patients who received at least 4 cycles of ^177^Lu-PSMA treatment, nearly a quarter of patients experienced a severe decrease in GFR 12 months after the treatment. In the following 2-3 years, some patients experienced further deterioration of renal function (30). However, due to the small number of patients, the practicality of these data is limited. Sathekge MM et al. (24) reported a minimal rise in the number of patients with renal toxicity after ²²⁵Ac-PSMA RLT. In their study, 22 (5%) patients were seen with grade 3 or higher renal toxicity. Perhaps due to the short follow-up period, our investigation demonstrated no substantial renal toxicity and just found a slight decline in GFR following therapy. Therefore, although acute toxicity (e.g.,xerostomia) are controllable, long-term follow-up highlights that cumulative hematological/renal toxicities are key issues. Long-term toxicity monitoring is crucial for optimizing the risk-benefit ratio, especially in patients with a longer survival time.

Our research also has some limitations. The study’s sample size is comparatively small, potentially introducing bias into the obtained data and conclusions. In the future, we will expand the sample size through multi-center collaboration to enhance the reliability of the conclusions. Because of the limited duration of the follow-up period and the inadequate amount of data available for collection, it was not feasible to assess the patient’s quality of life post-treatment, which may affect the comprehensive interpretation of the long-term effects of the treatment. Also, no control group was set in this study. Therefore, it is impossible to directly compare the efficacy differences between ^225^Ac-PSMA-617 and other treatment options (such as ^177^Lu-PSMA or chemotherapy). In the future, a prospective randomized controlled trial is planned to further verify the advantages of this treatment.

## Conclusion

^225^Ac-PSMA-617 has shown encouraging results in mCRPC patients, with good disease control rates and low side effects even when other treatment methods are no longer effective. Future research should prioritize conducting high-quality, multicenter, and prospective randomized controlled trials with several groups to thoroughly investigate the effectiveness and safety of various disease stages and combination therapy for prostate cancer.

## Data Availability

The original contributions presented in the study are included in the article/**Supplementary Material**, further inquiries can be directed to the corresponding author/s.
