# Supplementary material for: The efficacy and safety of 225Ac-PSMA-617 in metastatic castration-resistant prostate cancer
Source: Front Oncol. 2025 Feb 28;15:1516860. doi: 10.3389/fonc.2025.1516860 (PMC11906306; doi:10.3389/fonc.2025.1516860)

Supplementary table:

Table 5:The Molecular Response after ^225^Ac-PSMA-617

|  | The dose  ^225^Ac-PSMA-617 | RECIST 1.1 | PERCIST 1.0 |
| --- | --- | --- | --- |
| Patient 2 | 2 | PD | PMD |
| Patient 3 | 2 | PD | PMD |
| Patient 4 | 1 | SD | PMR |
| Patient 5 | 1 | SD | PMR |
| Patient 6 | 1 | PR | PMR |
| Patient 7 | 1 | PD | PMD |
| Patient 8 | 2 | PD | PMD |
| Patient 10 | 1 | SD | SMD |
| Patient 11 | 1 | PR | PMR |
| Patient 13 | 1 | SD | SMD |
| Patient 15 | 4 | PR | PMR |
| Patient 16 | 2 | SD | SMD |
| Patient 17 | 2 | PD | PMD |
| Patient 20 | 4 | PR | PMR |
| Patient 21 | 1 | SD | PMR |
| Patient 23 | 4 | PD | PMD |
| Patient 26 | 1 | PD | PMD |
| Patient 29 | 3 | SD | PMR |

PD:progressive disease; PMD:progressive metabolic disease; SD:stable disease;SMD:stable metabolic disease; PR:partial response; PMR:partial metabolic response.

Table 6: the PSA at baseline and after ^225^Ac-PSMA RLT of the patients experienced this "PSA flicker"

| patient  PSA(ng/ml) | at baseline | after the first cycle | after the second cycle | after the third cycle |
| --- | --- | --- | --- | --- |
| Patient 1 | 291.157 | 471 | 87.5 | / |
| Patient 2 | 601.326 | 1058.948 | 887.72 | 454.02 |

Supplementary figures:

Figure 4. Overall survival(OS) after treatment with ^225^Ac-PSMA-617


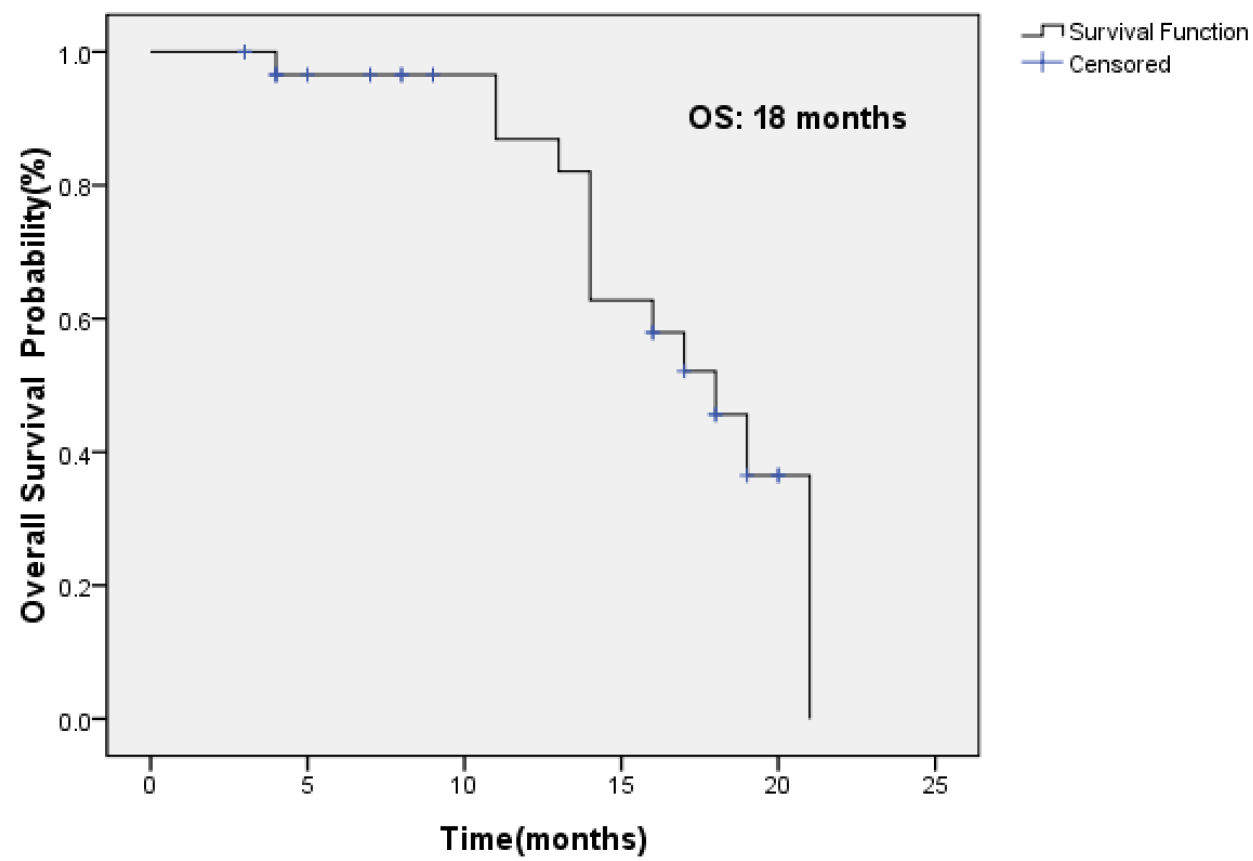


Figure 5. Progression free survival(PFS) after treatment with ^225^Ac-PSMA-617


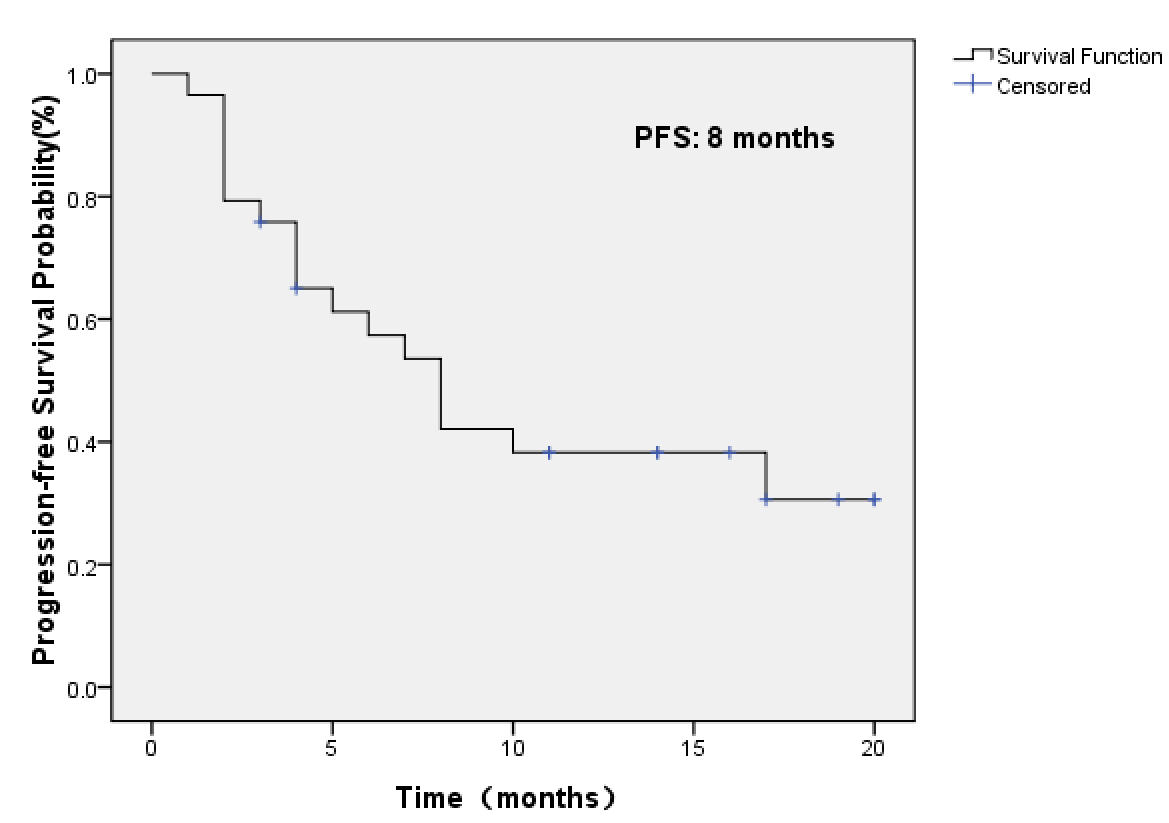


Figure 6. Progression free survival (PFS) associated with any PSA response


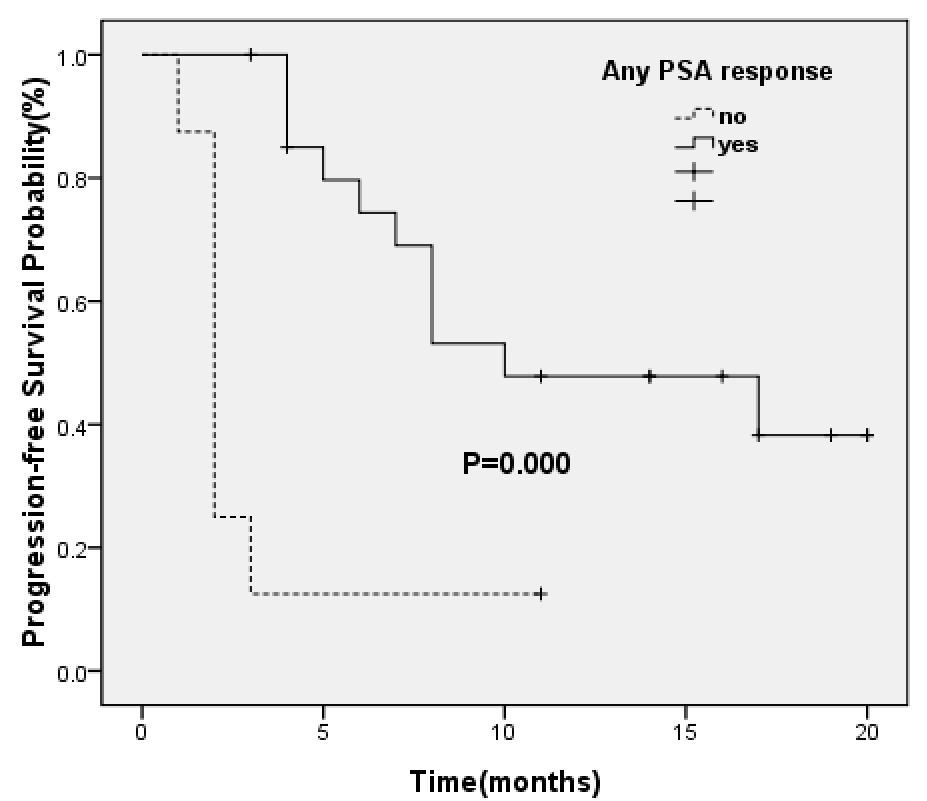


Figure 7. Progression free survival (PFS) between disease control and disease progression.


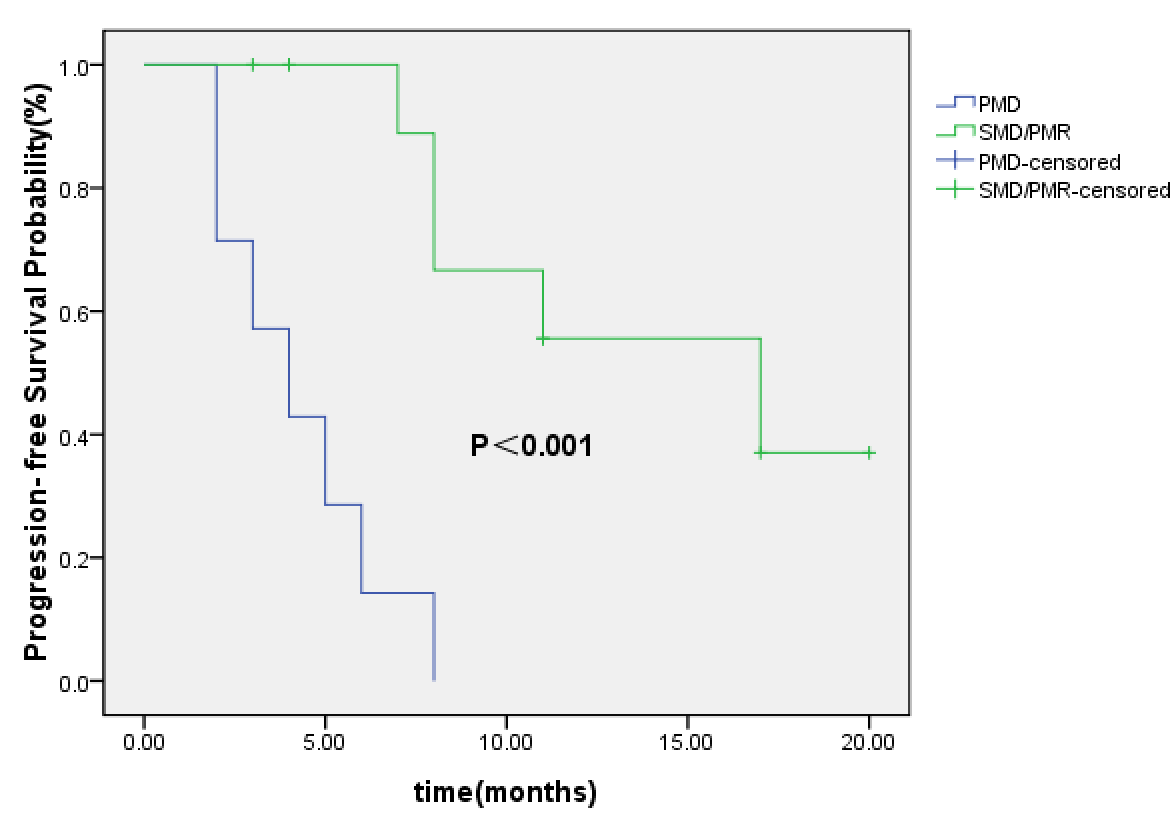


Figure 8. Laboratory test results during baseline and after treatment


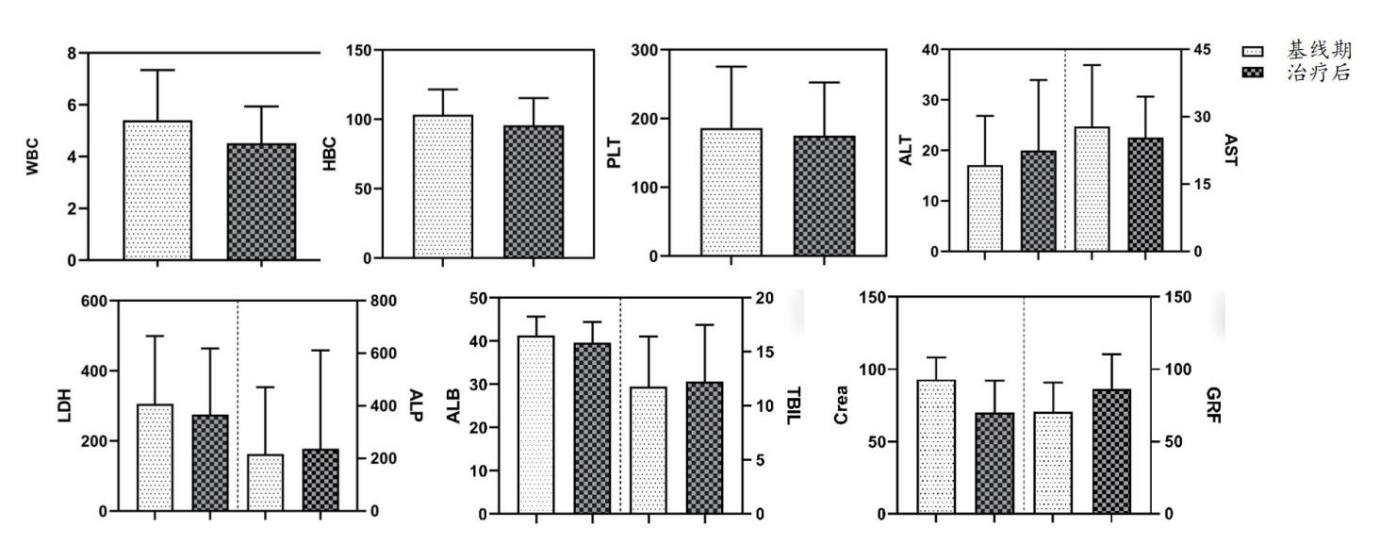


Patient 2: After the first cycle of ^225^Ac-PSMA RLT, the PSA increased from 601.326 ng/ml to 1058.948 ng/ml,but a continuous decline in PSA during subsequent treatments.Finally, after the third cycle treatment, the PSA decreased to 454.02 ng/ml. After two cycles of ^225^Ac-PSMA RLT, the expression of PSMA in most lesions was significantly decreased, as shown in Figure 9-10.

Figure 9. ^68^Ga-PSMA-11 PET/CT at baseline of patient 2.


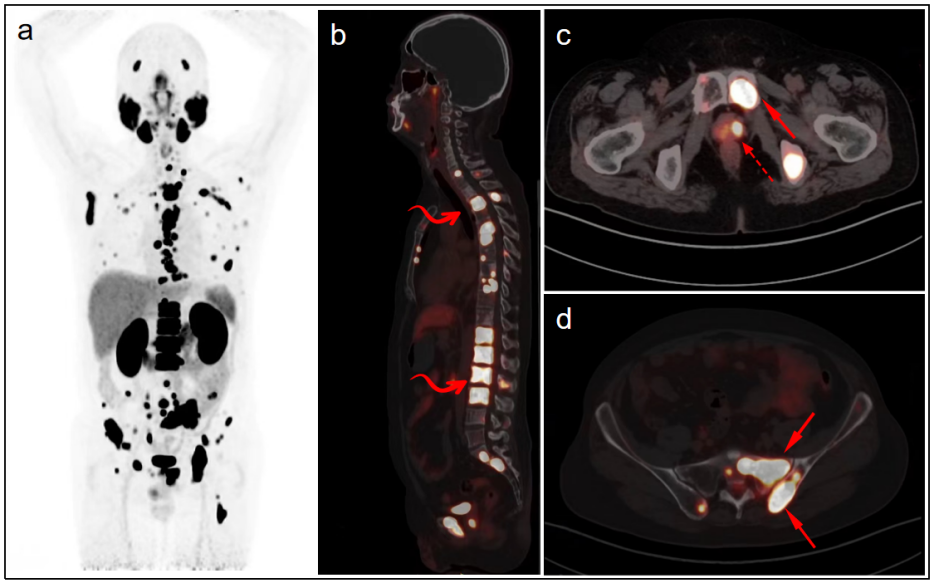


Figure10. ^68^Ga-PSMA-11 PET/CT afther two cycles of ^225^Ac-PSMA RLT of patient 2.


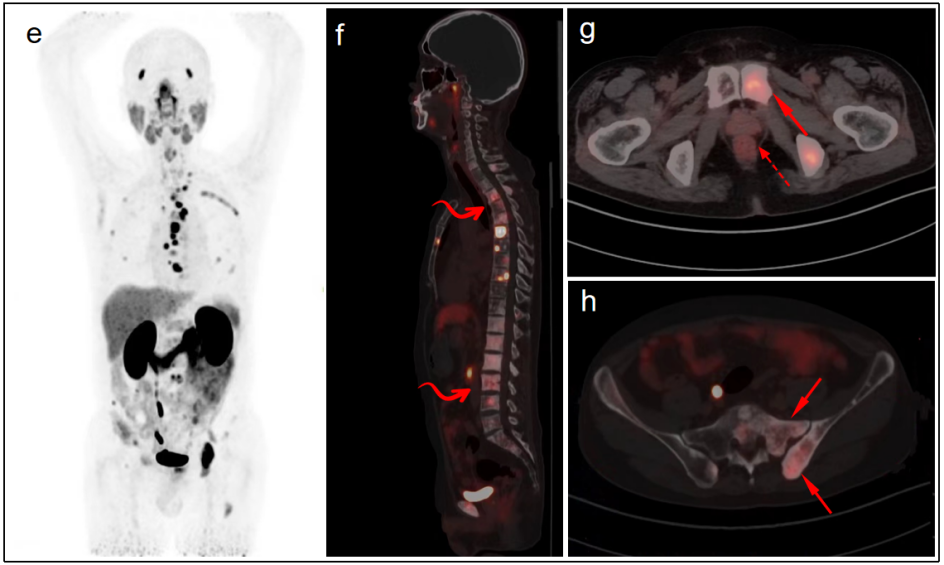

Supplement: Supplementary file 1 [file DataSheet1.docx]
